# Identification of High-Risk Human Papillomavirus DNA, p16, and E6/E7 Oncoproteins in Laryngeal and Hypopharyngeal Squamous Cell Carcinomas

**DOI:** 10.3390/v13061008

**Published:** 2021-05-27

**Authors:** Andrejs Lifsics, Valerija Groma, Maksims Cistjakovs, Sandra Skuja, Renars Deksnis, Modra Murovska

**Affiliations:** 1 Department of otolaryngology, Riga Stradiņš University, Pilsoņu 13, LV-1002 Riga, Latvia; 2 Institute of Anatomy and Anthropology, Riga Stradiņš University, Kronvalda blvd 9, LV-1010 Riga, Latvia; valerija.groma@rsu.lv (V.G.); Sandra.Skuja@rsu.lv (S.S.); 3 Institute of Microbiology and Virology, Riga Stradiņš University, Rātsupītes 5, LV-1067 Riga, Latvia; maksims.cistjakovs@rsu.lv (M.C.); modra.murovska@rsu.lv (M.M.); 4 Department of Head and Neck Surgery, Oncology Centre of Latvia, Riga Eastern University Hospital, Hipokrāta 2, LV-1038 Riga, Latvia; renars.deksnis@gmail.com

**Keywords:** HPV, larynx, hypopharynx, squamous cell carcinoma, PCR, immunohistochemistry, p16 regulatory protein, E6/E7 viral oncoproteins

## Abstract

Human papillomavirus (HPV) was proven to play a significant role in cancer development in the oropharynx. However, its role in the development of laryngeal (LSCC) and hypopharyngeal squamous cell carcinoma (HPSCC) remains to be clarified. High-risk HPV (HR-HPV) viral proteins E6 and E7 are considered to be pertinent to HPV-related carcinogenesis. Hence, our aim was to estimate LSCC and HPSCC for HR-HPV DNA, p16, and E6/E7 oncoprotein status by using molecular virology and immunohistochemistry methods. The prevalence of HPV16 infection was 22/41 (53.7%) and 20/31 (64.5%) for LSCC and HPSCC, accordingly. The majority of HPV16+ tumor samples were stage III or IV. In most samples, the presence of either HPV16 E6 or HPV16 E7 viral protein in dysplastic or tumor cells was confirmed using immunohistochemistry. Our results suggest a high prevalence of HPV16 as a primary HR-HPV type in LSCC and HPSCC. The lack of HPV E6/E7 oncoproteins in some tumor samples may suggest either the absence of viral integration or the presence of other mechanisms of tumorigenesis. The utilization of p16 IHC as a surrogate marker of HR-HPV infection is impractical in LSCC and HPSCC.

## 1. Introduction

Laryngeal and hypopharyngeal cancers belong to a large group of squamous cell carcinomas of the head and the neck. The annual incidence of these types of tumors in the world population exceeds 250,000 new registered cases [1]. Among these two anatomically closely related cancers, hypopharyngeal tumors are known to be associated with worse outcomes [2].

In the last three decades, the contribution of HPV to the development of oropharyngeal squamous cell carcinoma (OPSCC) was proven [3,4]. Furthermore, HPV-associated OPSCC is reported as biologically and clinically distinct from tobacco and alcohol-related OPSCC [5]. A better prognosis of HPV-positive (HPV+) OPSCC, compared to HPV-negative (HPV-) OPSCC, with 80% or higher three year overall survival for locally advanced disease was confirmed [3,6,7,8,9]. Currently, the biological role of HPV in the pathogenesis of laryngeal and hypopharyngeal squamous cell carcinomas (LSCC and HPSCC) appears controversial and has not been sufficiently studied [10,11].

Accumulating evidence suggests the epithelium of skin and mucosa is infected through superficial defects, and, upon establishment of viral genomes in the nucleus of infected cells as episomes, the early viral genes E1, E2, E6, and E7 are expressed. Furthermore, HPV episomes are maintained in poorly differentiating but actively dividing basal epithelial cells by replicating along with cellular DNA [4,12,13]. Along with the natural upward migration and the further differentiation of epithelial cells, the productive phase of the viral life cycle is triggered, allowing for the continued expression of E6 and E7 in differentiating cells [14]. Therefore, from an oncogenic standpoint, high-risk HPV (HR-HPV) E6 and E7 proteins are of the utmost importance [15].

While the viral oncoprotein E6 induces the degradation of p53, leading to the inhibition of elimination by apoptosis in affected epithelial cells, oncoprotein E7 inactivates tumor suppressor proteins of the pRb family, promoting the transcription of p16 [5,13,15,16,17]. Upon proteasomal degradation of pRb, p16 becomes overexpressed and, therefore, applicable for immunohistochemical (IHC) detection of HPV-driven tumors [18,19]. The necessity of the inclusion of p16 status when diagnosing OPSCC was confirmed by updating a TNM Classification of Malignant Tumors, and p16 IHC proved to be a reliable and, therefore, stand-alone test for the detection of HPV in OPSCC [5,20]. Furthermore, the eighth edition of the AJCC Cancer Staging Manual separated HPV positive OPSCC from HPV negative OPSCC, highlighting the biological role and the prognostic significance of p16 [21,22]. However, the question of whether HPV infection actively contributes to cancer development needs a substantial examination [23]. The presence of the p16-positive (p16+) OPSCCs in HPV-cases was demonstrated by previous studies, thus suggesting the existence of other mechanisms of p16 overexpression [24,25,26]. Simultaneously, contrary conditions demonstrating the presence of p16-negative (p16-) but HPV RNA-positive tumors were reported [27]. This is the reason for the suggested multimodality testing for OPSCC—both p16 and HPV DNA/ RNA detection [28].

Application of p16 for the assessment of transcriptionally active HR-HPV infection in non-OPSCCs and discussion around it highlight the complexity arising when exploring LSCC and HPSCC [29,30,31]. Currently, it is recommended that HPV testing on head and neck cancers should be limited to assays for HR-HPV types, and it should be routinely performed on (but also limited to) OPSCC and metastatic SCCs in neck lymph nodes from unknown primary sites [24,30,31]. The prevalence of HPV+ HPSCC and LSCC varies depending on region and study center reports, suggesting 5–20% of laryngeal cancers and as little as 0% of hypopharyngeal cancers are associated with HR-HPV infection [32,33,34,35]. Previous studies validated HPV-specific testing modalities such as HPV DNA-ISH, DNA polymerase chain reaction (PCR), mRNA RT PCR, and mRNA ISH for viral oncoproteins E6 and E7 as well as p16 IHC, including those performed on formalin-fixed, paraffin-embedded (FFPE) specimens in OPSCC; however, more clarity is needed to better explore these tests applicable to LSCC and HPSCC cases [36].

This study aimed to estimate LSCC and HPSCC for HR-HPV DNA, p16, and E6/E7 oncoprotein status by using molecular virology and immunohistochemistry methods.

## 2. Materials and Methods

### 2.1. Patients’ Characteristics

Seventy-two patients, 68 (94.4%) males (median age 64.9 (range 44.2–83.3)) and 4 (5.6%) females (median age 70.8 (range 53.5–77.5)) with histologically confirmed LSCC and HPSCC, treated at the Latvian Oncology Centre between January 2015 and August 2019, were enrolled in the study.

The clinical data of patients included information on TNM stage, smoking and drinking habits, and clinical features of the disease at the time of presentation. Forty-one of 72 patients had LSCC; for 31 patients, the primary tumor site was the hypopharynx. Most patients (88.9%) were smokers; 15 (20.8%) were heavy drinkers [37]. The patients’ data are summarized in Table 1.

The study was approved by the Ethical Committee of Riga Stradiņš University (Decisions No. 3/24.09.2015.) and conducted according to the Declaration of Helsinki.

### 2.2. DNA Extraction

Total DNA was extracted from either fresh frozen biopsies and surgical materials (34 LSCC and 3 HPSCC) or FFPE tumor tissue blocks (28 HPSCC and 7 LSCC).

DNA extraction from the fresh frozen tumor tissue samples was carried out with the phenol/chloroform extraction method.

DNA extraction from FFPE tumor samples was carried out with the blackPREP FFPE DNA Kit (Analytik Jena, Germany) following the manufacturer’s protocol. Three to six 10 µm thick sections cut from FFPE samples were used for DNA extraction. Each sample was sectioned separately with a sterile blade to exclude cross-contamination of specimens.

The concentration and the quality of the extracted DNA were measured spectrophotometrically (Nanodrop ND-1000 Spectrophotometer, Thermo Fisher Scientific, Waltham, MA, USA). Beta- (β-) globin PCR with appropriate primers was used to determine the quality of isolated DNA [38]. All samples were β-globin positive.

### 2.3. HPV DNA Detection Using MY09/11 and GP5+/6+ Consensus Primers

Initially, separate regular polymerase chain reactions (PCR) with consensus primers MY9/MY11 and GP5+/6+ were used for the detection of a broad range of HPV types [39,40]. The results were visualized using 1.7% ethidium bromide electrophoreses gel. The amplification products of 450 base pairs (bp) and 150 bp length for MY09/11 and GP5+/6+, correspondingly, were considered HPV-positive (Table 2). Positive and negative controls were included in each reaction.

### 2.4. HPV Genotyping

Two types of primers were used: the type-specific primers for HPV 16 and 18 (L1) and the Anyplex II HPV28 multiplex real-time-PCR (RT-PCR).

Genomic sequences of HPV16 and HPV18 type-specific primers’ are summarized in Table 2 [40]. Amplimers of 152 bp in length were produced by HPV16 primers, whereas 216 bp long amplimers were produced by HPV18 primers. The results were visualized by electrophoresis in 1.7% agarose gel. Each reaction included positive and negative controls.

Anyplex II HPV28 multiplex RT-PCR was performed as recommended by the manufacturers (Seegene, South Korea). In total, 5 μL of specimen DNA were added in each of the two sets (wells) with 20 μL PCR reaction mix. Set A consisted of a primer mix for 14 HR-HPV types (HPV16, 18, 31, 33, 35, 39, 45, 51, 52, 56, 58, 59, 66, and 68), and set B consisted of a primer mix for five possible HR-HPV types (HPV26, 53, 69, 73, and 82) and nine LR- HPV types (HPV6, 11, 40, 42, 43, 44, 54, 61, and 70). Both primer sets were designed for the HPV L1 gene and produced 100 and 200 bp amplicons, accordingly. Melting curves allowing the semiquantitative assessment and the differentiation between high (+++), moderate (++), or low (+) viral load were obtained at 30, 40, and 50 cycles and had positive and negative internal controls.

The kit had DNA quality control detecting the β-globin gene; all analyzed samples were β-globin positive. The results were analyzed using the Seegene Viewer software (Seegene, South Korea).

### 2.5. Immunohistochemistry

LSCC specimens were obtained during laryngectomy and cordectomy. A small piece of surgically obtained LSCC was further processed for molecular testing, whereas the remaining material as a FFPE tissue was submitted to IHC.

In contrast, HPSCC specimens were mostly obtained during a biopsy procedure and further processed as FFPE tissue samples at Latvian Oncology Centre. Only tumor tissue samples confirmed by histopathological examination as HPSCC were used in the study.

HPV 16 E6/E7 proteins and p16 were assessed immunohistochemically. Histological sections of 4–5 µm were cut from FFPE tissues and mounted on slides. The consecutive sections were used as negative controls of the immunohistochemical reactions and for hematoxylin and eosin (H&E) staining to confirm the diagnosis. Immunohistochemistry (IHC) was performed manually using sections collected on SuperFrost Plus slides (Gerhard Menzel GmbH, Germany). Immunostaining was carried out following the previously used IHC protocol [41,42].

The sections were incubated at 4 °C overnight with the following primary antibodies: monoclonal mouse anti-CDKN2A/p16INK4a antibody (Abcam, Cambridge, UK, 1:300 dilution, ab201980); monoclonal mouse anti-HPV16 E6 + HPV18 E6 antibody (Abcam, Cambridge, UK, prediluted, ab51931), which recognize the HPV early antigen E6 of HPV 16 and 18 [43,44,45]; mouse monoclonal anti-HPV16 E7 antibody (Santa Cruz Biotechnology, Inc., Santa Cruz, CA, USA, 1:50 dilution, sc-6981).

The amplification of the primary antibody and the visualization of reaction products were performed by applying the HiDef Detection HRP Polymer system and diaminobenzidine tetrahydrochloride substrate kit (Cell Marque, Rocklin, CA, USA). The sections were counterstained with Mayer’s hematoxylin, washed, mounted, and covered with coverslips. The immunohistochemical controls included the omission of the primary antibody. The assessment of immunostaining was performed by two independent observers blinded to clinicopathological data.

The sections were photographed by a Leitz DMRB bright-field microscope using a DFC 450C digital camera or scanned with a Glissando Slide Scanner (Objective Imaging Ltd., Cambridge, UK) with a 10×, 20×, and 40× objective.

Cells that were labeled with anti-CDKN2A/p16INK4a, anti- HPV16 E6 + HPV18 E6, and anti-HPV16 E7 antibody and that displayed brown reaction products were considered immunopositive.

The assessment of immunostaining of p16 was carried out by determining positive vs. negative structures with a cut-off at 50% tumor cells independently of the reaction proposed by Hong et al., (2013) [46]. The immunostaining assessment for E6 and E7 viral proteins was performed semiquantitatively in 20 randomly selected visual fields of each sample (magnification 400×) representing the tumor and the surface epithelium of the regions of interest. The levels of E6 and E7 were graded as negative—0%, weak—≤10%, moderate—11–50%, and strong—>50%, respectively.

### 2.6. Immunofluorescence

To better visualize the cellular distribution and the localization of the HR-HPV16 E7 oncoprotein, the tumor tissue specimens were processed for fluorescence-based immunodetection. The sections immunoreacted with mouse monoclonal anti-HPV16 E7 antibody (Santa Cruz Biotechnology, Inc., Santa Cruz, CA, USA, 1:50 dilution, sc-6981) overnight at 4 °C were washed in PBS and incubated with goat anti-mouse IgG-FITC: sc-2010 (Santa Cruz Biotechnology, Inc., Santa Cruz, CA, USA 1:300) as the secondary antibody. Then, sections were counterstained with 4′,6-diamidino-2phenylindole (DAPI) (Thermo Fisher Scientific, Invitrogen, Renfrew, UK, 1:3000) and mounted in Prolong Gold with DAPI (Thermo Fisher Scientific, Waltham, MA, USA). Imaging was performed using an Eclipse Ti-E confocal microscope (Nikon, Tokyo, Japan).

The workflow of the present study is summarized in Appendix A.

### 2.7. Statistical Data Analysis

All the statistical analyses were performed using the GraphPad Prism 9 (demo, GraphPad Software, La Jolla, CA, USA). Anderson–Darling, D’Agostino and Pearson, and Shapiro–Wilk normality tests were applied to assess numerical data distribution. The comparison of means between different groups of numerical variables was performed using one-way ANOVA. For data with a non-Gaussian distribution, Kruskal–Wallis or Friedman’s test (for paired groups) followed by the two-stage step-up method of Benjamini, Krieger, and Yekutieli as a false discovery rate controlling test were used. To compare numerical values between two groups, the Mann–Whitney test or the Wilcoxon test (for pared groups) was applied. The relations between the analyzed groups were investigated using nonparametric Spearman’s correlation analysis [47]. The IHC results were expressed as violin plots and stacked bar graphs, and a *p*-value of less than 0.05 (*p* < 0.05) was considered statistically significant.

## 3. Results

### 3.1. HPV DNA Detection Using MY09/11 and GP5+/6+ Consensus Primers

Eleven (15.3%) out of 72 tumor samples were positive for HPV genomic sequences using PCR with MY9/11 consensus primers. In turn, 55 (76.4%) samples demonstrated positivity using PCR with GP5+/6+ primers. By summing up, when tested with consensus PCRs, 61 (84.7%) tumor tissue samples were found positive for HPV DNA—31 HPSCC and 30 LSCC samples.

### 3.2. HPV Genotyping Using HPV16 and HPV18 L1 Primers

All the tumor tissue samples (*n* = 72) were subjected to HPV genotyping using HPV16 and HPV18 L1 primers. A total of 2/72 tumor tissue samples (both LSCC), positive when detected by HPV16 L1 primers, were negative in consensus PCRs. No specific HPV-18 genomic sequence was found in any of the samples.

Overall, 26/72 (36.1%) samples were positive for HPV16—10 LSCC and 16 HPSCC samples. In total, 63 HPV+ samples were considered applicable for further analysis; among them, 61 were selected using consensus PCRs, whereas 2 additional samples were selected by HPV16 L1 PCR.

### 3.3. Detection of HPV Using Anyplex II HPV28 RT-PCR

All 63 HPV-positive samples confirmed using either consensus primers or HPV16-specific primers were further explored by Anyplex II HPV28 multiplex RT-PCR. All samples were β-globin positive (internal control). When assessed by Anyplex II HPV28 multiplex RT-PCR, 28/63 samples were HPV-negative. HPV16 monoinfection was confirmed in 32/63 samples, whereas HPV16 and HPV31 coinfection was confirmed in 2/63 samples, and HPV16 and HPV56 coinfection in 1/63 samples. When HPV+ samples were stratified by the location, 19 LSCC and 13 HPSCC presented as HPV16+, 2 LSCC presented as demonstrating HPV16 and HPV31 coinfection, and 1 HPSCC presented as demonstrating HPV16 and HPV56 coinfection.

**Figure 1:**
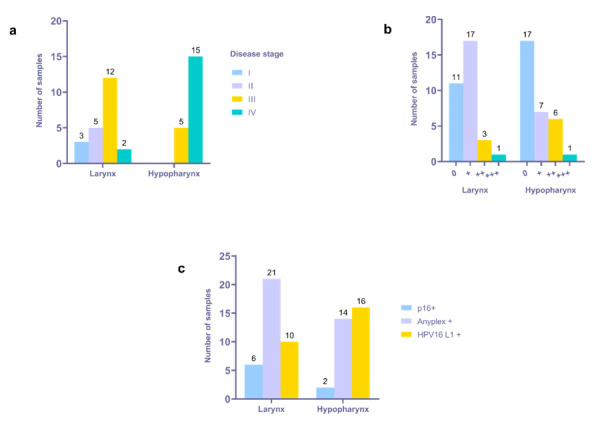
Distribution of HPV16+ tumor samples according to location, disease stage, and PCR data. (**a**) Distribution of HPV16+ tumor samples according to location and disease stage. (**b**) Distribution of HPV16+ samples according to location and Anyplex assay results; 0—negative, +—low viral load, ++—moderate viral load, +++—high viral load. (**c**) Cross-reference of p16 and HPV status.

Interestingly, seven (one LSCC and six HPSCC) HPV16+ tumor tissue samples, confirmed by applying HPV16 L1 PCR, were negative according to Anyplex II HPV28 RT-PCR, thus contributing to a total number of 42/72 (58.3%) HPV16+ samples. The prevalence of HPV16 infection, including multiple infections in a sample, was 22 of 41 (53.7%) for LSCC and 20 of 31 (64.5%) for HPSCC (Supplementary Table S1). Most of the HPV16+ samples were stage III or IV tumors (Figure 1a).

Twenty-one HPV16+ samples presented with low, nine with moderate, and two with high viral load, respectively, when detected using the Anyplex II assay. The presence of multiple HPV infections was confirmed in three samples (one HPSCC and two LSCC). A low viral load for both HPV types was established in the HPSCC sample presented with HPV16 and 56 coinfections. Similarly, a low viral load for both HPV types was confirmed in the LSCC sample presented with HPV16 and 31 coinfections. By contrast, a low viral load was confirmed for HPV16, whereas a high viral load was confirmed for HPV31, assessed in the remaining LSCC sample with HPV 16 and 31 coinfections, thus suggesting the dominance of HPV31. The HPV16 RT-PCR data are summarized in Figure 1b.

### 3.4. Immunohistochemical Detection of p16^INK4a^

Immunohistochemically, the expression of p16INK4a was confirmed in 11.1% of the tumor tissue samples. Out of 41 LSCC and 31 HPSCC, in six and two samples, respectively, were found p16+. Cross-referencing p16 and HPV status, the tumors were stratified as follows: 7/72 (9.7%)—p16+/HPV+, 1/72—p16+/HPV-, 8/72 (11.1%)—p16-/HPV-, and 56/72 (77.8%)—p16-/HPV+. Most of the p16+/HPV+ tumors were LSCC (*n* = 6), whereas two were HPSCC (Figure 1c). The only p16+/HPV- tumor was LSCC. Six out of seven p16+/HPV+ tumors had HPV16 mono-infection, whereas one had HPV16 and 31 coinfections. A total of 35 out of 56 p16-/HPV+ tumors, 27 LSCC and 29 HPSCC, were positive for HPV16 when explored by Anyplex II RT-PCR and HPV16 L1 primers’ PCR, whereas two had the aforementioned HPV coinfections.

### 3.5. Immunohistochemical Detection of HPV16 E6 and E7 Oncoproteins in LSCC and HPSCC

The immunohistochemical detection of HPV16 oncoproteins E6 and E7 in 42 FFPE (22 LSCC and 20 HPSCC) samples was based on the primary recognition of HPV16 as the main HPV type when applying molecular virology assays.

**Figure 2:**
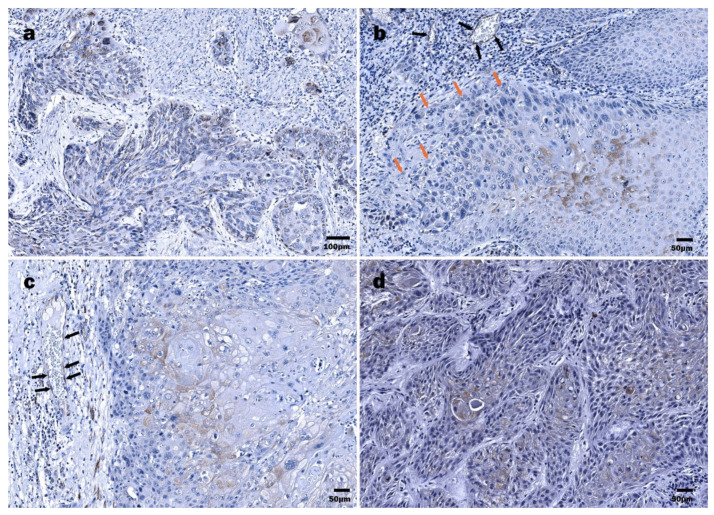
Immunohistochemical detection of HPV16/18 E6 oncoprotein: (**a**) LSCC, tumor cords and nests composed of diffusely distributed E6 protein-positive cells interspersed by the E6 oncoprotein-negative cells; (**b**) LSCC, differentiated suprabasal tumor cells demonstrating abundant HPV16 E6-positive cytoplasm and polymorphous nuclei (orange arrows), E6-positive endotheliocytes (black arrows) within a tumor stroma; (**c**) LSCC, HPV16/18 E6 positivity in suprabasal, more differentiated tumor cells; E6-positive endothelial cells (black arrows); (**d**) HPSCC, densely packed tumor cords demonstrating HPV16 E6 oncoprotein positivity, almost exclusively in more differentiated cells.

**Figure 3:**
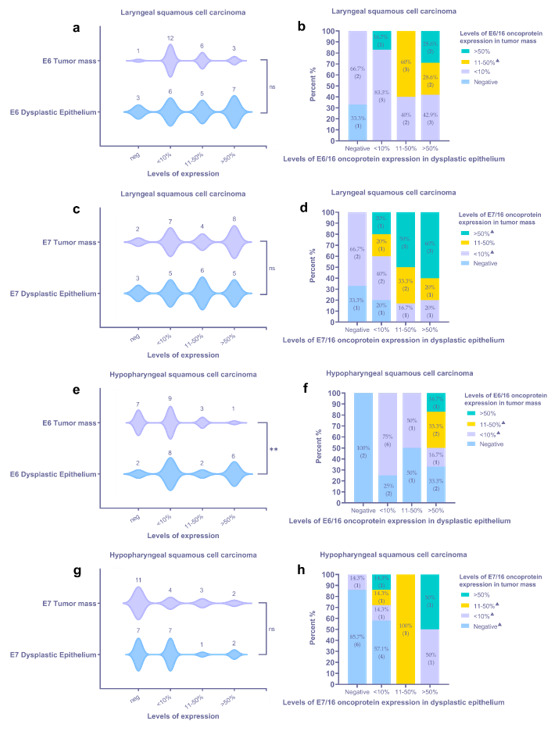
Assessment of viral oncoproteins E6 and E7 in HPV16+ laryngeal (**a**–**d**) and hypopharyngeal (**e**–**h**) tumor tissue samples using IHC and statistics: (**a,c**) characterization of HPV oncoprotein E6 (**a**) and E7 (**c**) immunoexpression within a tumor mass and dysplastic epithelium of LSCC samples; (**b**,**d**) the IHC expression levels for HPV oncoprotein E6 (**b**) and E7 (**d**) in a tumor mass assessed in relation to the levels in a dysplastic epithelium of the corresponding LSCC sample; (**e**,**g**) characterization of HPV oncoprotein E6 (**e**) and E7 (**g**) immunoexpression within a tumor mass and dysplastic epithelium of HPSCC samples; (**f**,**h**) the IHC expression levels for HPV oncoprotein E6 (**f**) and E7 (**h**) in a tumor mass assessed in relation to the levels in a dysplastic epithelium of the corresponding HPSCC sample. Violin plots: asterisks represent a significance level (ns—non-significant, * *p* < 0.05, ** *p* < 0.01) of differences between groups (two-tailed Wilcoxon test); stacked bar graphs—crosstab analysis, triangles (^▲^) represent a sample lacking an epithelial region suitable for assessment and, therefore, were excluded from crosstab analysis.

The expression of E6 in HPV16+ LSCC specimens was detected in 21 of 22 cases. The immunoreactive structures were revealed both within the tumor mass and the surface epithelium of the region of interest, demonstrating dysplastic features. One specimen contained only the tumor nest. In 3/22 cases, immunoexpression of HPV16/18 E6 oncoprotein in the tumor mass was strong (>50%); furthermore, among them, 2/22 cases presented with strong positivity in a dysplastic epithelium (Figure 2a and Figure 3a,b).

In most LSCC samples (12/22), the expression of the E6 oncoprotein in the tumor mass was low (Figure 3a). By contrast, a dysplastic epithelium demonstrated an almost equal distribution of expression levels; six, five, and seven cases presented with levels <10%, 11–50%, and >50%, respectively. In 3/22 cases, dysplastic epithelial cells were E6-negative; however, among three tumors, two neoplasms presented with low immunopositivity (Figure 3b). In most specimens, positive staining in the invasive front of tumor mass was noticed, commonly presented as the decoration of the suprabasal cells (Figure 2c). Furthermore, the expression of the HPV E6 viral protein in the endothelial cells of small blood vessels was demonstrated (Figure 2b,c).

**Figure 4:**
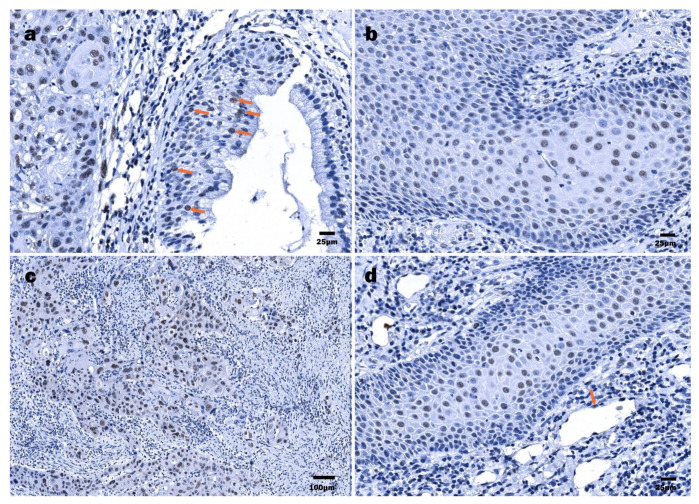
Immunohistochemical detection of HPV16 E7 oncoprotein: (**a**) LSCC, tumor cells within a nest and some surface cells (orange arrows) demonstrating nuclear HPV16 E7 positivity; (**b**) LSCC, numerous HPV16 E7-positive cells displaying nuclear immunostaining pattern; (**c**) LSCC, highly polymorphous HPV16 E7-positive tumor cells demonstrating nearly total nuclear decoration; (**d**) HPSCC, numerous HPV16 E7-positive cells displaying nuclear immunostaining pattern, endothelial (orange arrow) cells.

HPV16 E7 protein immunoexpression was confirmed in 20/21 LSCC specimens (Figure 3c). In two cases, the specimen did not contain an epithelial region, being predominantly tumor. In one case, there was not enough material for suitable immunohistochemical detection of the HPV16 E7 protein. Cells labeled by the HPV16 anti-E7 antibody commonly displayed a nuclear staining pattern and rarely showed nuclear and cytoplasmatic staining pattern. The presence of HPV16 E7 oncoprotein expression was confirmed in an affected epithelium, both stratified squamous and pseudostratified ciliated (Figure 4a). Dominant immunostaining was confirmed in basal and suprabasal cells (Figure 4b). Strong immunoexpression of the HPV16 E7 oncoprotein in the tumor nests was found in 8/21 LSCC samples (Figure 3c,d and Figure 4c). Finally, HPV16 E7 oncoprotein immunopositivity was demonstrated along an intimal aspect of small blood vessels.

Eighteen out of 20 HPSCC samples were HPV16 E6 oncoprotein positive. However, immunohistochemically HPV16 E6 positivity in a tumor mass was confirmed in 13/20 cases (Figure 3e,f). In most samples, cytoplasmatic immunoexpression of the HPV16 E6 oncoprotein within the dysplastic epithelium was evidenced. Commonly, the expression of the HPV16 E6 oncoprotein within a tumor mass was low. Immunostaining within differentiated tumor cells was also demonstrated (Figure 2d). Similar to the LSCC samples, some endothelial cells were found to be HPV16 E6-positive.

An even smaller number of HPV16 E7 oncoprotein positive HPSCC cases were found when compared to HPV16 E6-positive cases, represented by 13/20 cases (Figure 3g,h). Furthermore, a positive reaction within a tumor mass was confirmed in 9/20 cases. Among them, a strong tumor immunoreaction was demonstrated in 2/20 HPSCC samples. The expression was nuclear only. In differentiated suprabasal cells and endothelial cells, a positive HPV16 E7 oncoprotein reaction was found (Figure 4d).

Collectively, the immunoexpression of HPV16 E6/E7 oncoproteins within a tumor mass was not confirmed in 7/42 samples (one LSCC and six HPSCC). Five of these samples were HPV16+ with the Anyplex RT-PCR and two with HPV16 L1 PCR, all of them p16-. Immunohistochemically, only one HPSCC and one LSCC sample were both HPV16 E6 and E7 oncoprotein negative; simultaneously, the HPSCC sample was HPV16+ by HPV16 L1 PCR and the LSCC sample by Anyplex RT-PCR. Matched HPV16 E6/E7 positivity was demonstrated in 2/42 samples within the dysplastic epithelium. No significant differences in tumoral or dysplastic epithelial HPV16 E6/E7 oncoprotein expression were found, except a significant difference demonstrated for E6 oncoprotein positivity in the HPSCC samples (Figure 3e). Overall, a similar tendency of HPV oncoprotein E6/E7 expression was demonstrated within a tumor mass and the dysplastic epithelium in both LSCC and HPSCC (Figure 3a,c,g).

The results of semiquantitative RT-PCR and IHC E6/E7 oncoprotein immunoexpression were further submitted to nonparametric correlation analysis. A moderate positive correlation (r_S_ = 0.445, *p* = 0.056) between semiquantitative RT-PCR and HPV16 E7 IHC data was demonstrated in the LSCC tissue samples, particularly in the dysplastic epithelium. Additionally, there were weak-to-moderate positive correlations found in the HPSCC tissue samples; however, they failed to reach statistical significance (Supplementary Figure S1).

Additionally, we performed nonparametric correlation analysis of p16 and E6/E7 IHC, HPV16 L1 PCR, and Anyplex RT-PCR results. No statistically significant correlations were found in LSCC and HPSCC samples for the aforementioned data (Supplementary Figure S2).

**Figure 5:**
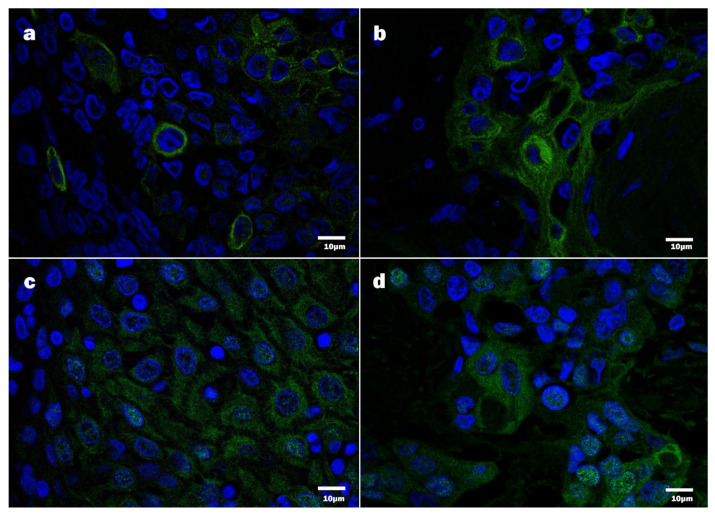
Detection of HPV16 protein E7 by immunofluorescence, confocal microscopy, DAPI—blue, HPV16 E7 immunopositive products—green: (**a**,**b**) HPV16 E7 positive tumor cells, displaying chiefly cytoplasmic positivity; (**c**,**d**) HPV16 E7 positive tumor cells, displaying cytoplasmic and nuclear positivity.

To ensure better visualization of the E7 oncoprotein, in addition to bright-field optics, fluorescence-based immunodetection was applied. By immunofluorescence, the presence of HPV16 E7 was confirmed in the cytoplasm and the nuclei of the tumor cells (Figure 5). Notably, nuclear E7 immunopositivity was confirmed and found to be in agreement with the bright-field optics observations; however, the intensity of staining greatly varied. The cytoplasmic E7 oncoprotein targeted was expressed in occasional or multiple cells constituting a tumor mass.

## 4. Discussion

According to the available data, around 20% of LSCC and 5% of HPSCC are attributable to HPV infection in the USA [48]. In Europe, the incidence of HPV+ head and neck cancer is lower [33]. However, it is higher in developed countries, such as the United Kingdom, Denmark, and Germany, than in less developed ones (mostly Eastern European countries) [49,50,51]. This difference may be explained by different lifestyles, preferences, and sexual habits. Notably, smoking, recognized as a significant factor influencing the development of head and neck cancer, is a common hazard in Latvian society [52]. However, the present study suggests a role of HPV in the carcinogenesis of non-oropharyngeal cancer and points at HPV16 being a predominant HPV type confirmed in LSCC and HPSCC. These results are consistent with the data published by other authors [33,53,54].

This study highlights the high incidence of HPV+ tumors and HR-HPV’s role in the pathogenesis of HPSCC and LSCC in Latvia when compared to Europe and North America. Higher prevalence of HPV16 + tumors—53.7% and 64.5% in LSCC and HPSCC, respectively, were demonstrated in the given study. However, the question of whether HPV infection in the tumor tissue is transcriptionally active needs further extensive investigation and remains open [55]. In this context, the detection of HPV E6/E7 mRNA in LSCC and HPSCC tissue samples may add clarity to this problem [56]. The other problematic issue is the necessity of better distinction between primary tumors and those that are an extension from different sites, for example, the oropharynx, which is generally considered to be most associated with HPV infection [33]. Often, in the late stages of the disease, it is hard to distinguish the primary tumor site. Therefore, optimization of diagnostic accuracy at the early and especially at the late stages of the malignant process is of pivotal significance. In this study, we confirmed the presence of HPV16 in a large portion of LSCC and HPSCC characterized as stage III and IV tumors, thus suggesting a possible linkage between the late stage of a tumor and a higher prevalence of HR-HPV infection. This evidence is in agreement with the results published by other authors reporting on the late stages of hypopharyngeal cancer presenting with a high prevalence of HR-HPV infection [57]. Further studies unraveling the intimate relationships between the stage of neoplasm and HPV status could be of interest.

To the best of our knowledge, few studies previously explored the presence of HPV oncoproteins E6 and E7 in tumor and dysplastic epithelial cells by IHC [58,59,60]. Previously, some HPV DNA and RNA in situ hybridization results obtained by other authors using FFPE samples and conventional light microscopy were reported [5,30,61]. In this study, FFPE samples selected from the HPV16+ tumors (*n* = 42) detected by molecular biology methods were used. Most HPV16+ samples demonstrated either oncoprotein E6/16 or E7/16 positivity. However, the absence of oncoprotein E6/E7 immunostaining, evidenced in some samples, is likely to suggest other, non-HPV-related mechanisms of tumor development.

The given study aimed to report on the peculiarities of tumorigenesis in the larynx and the hypopharynx and the likely differences between these two sites, highlighting HR-HPV DNA, p16, and E6/E7 oncoprotein status assessed using molecular virology and IHC methods. Even though most correlations failed to reach statistical significance, weak to moderate positive correlations between the molecular virology and the IHC results may indicate active HPV infection in these samples. However, the data about the activity of HPV infection (detection of viral mRNA) could clarify this hypothesis. In this study, PCRs confirmed the presence of HPV DNA in the LSCC and HPSCC samples; still, the molecular virology methods applied failed to distinguish between active and latent infection. On the other hand, the presence of HPV E6/E7 proteins, known as significant contributors to tumor development, suggests active participation of HR-HPV infection in tumorigenesis.

Interestingly, in some HPV16+ specimens, tumor cells stained negative for HPV16 E6/E7 oncoproteins, whereas dysplastic epithelium stained positive. Finally, some endothelial cells were found to be positive for HPV16 E6/E7 proteins. These results reflect the limitations of PCR assays, which do not specify the source of the genetic material. In general, the presence of HR-HPV E6 andE7 oncoproteins suggests a possibility of cancerous transformation of these cells. Viral integration and dysregulation of E6 and E7 gene expression is a common tumorigenesis mechanism confirmed in HPV-related cancers in general and in cervical cancer in particular [16,62]. However, in HPV-associated head and neck SCCs, viral integration occurs less regularly. In these tumors, dysregulation of the E6/E7 genes can be induced in the episomal state, for example, by the disruption of the HPV E2 binding sites by methylation [63,64,65]. The lack of HPV16 E6/E7 oncoproteins in the tumor cells and, contrarily, the appearance of these in dysplastic epithelial and endothelial cells demonstrated in our study may reflect the absence of HPV integration. In the advanced tumor stages, viral DNA could be cleared from the tumor itself. Other mechanisms of tumorigenesis could also exist.

Some authors suggested that HR-HPV infection may contribute to laryngeal carcinogenesis via integration of the viral DNA in the host cell genome and a further increase in p16 expression [66]. In this study, however, high numbers of p16-/HPV+ specimens in LSCC and HPSCC patients were demonstrated. Furthermore, there were no significant correlations found between the p16, E6/E7 IHC, and PCR data. These results are in agreement with other authors, suggesting p16INK4a can be used as a surrogate marker of HPV infection in OPSCC but appears impractical in laryngeal and hypopharyngeal cancers [31,67,68].

The use of broad spectrum of HPV-specific tests such as HPV DNA PCR, detection of HR-HPV and LR-HPV types, along with IHC staining of HPV surrogate marker p16 and viral oncoproteins E6/E7, confirmed by conventional and fluorescence-based immunodetection methods, may be considered as the strength of this study. A few limitations should be considered when interpreting our data. A moderate number of samples were used in this study. The second limitation is related to the absence of HPV mRNA data. These data would be of interest, bringing clarity to the question regarding the activity of HPV infection in analyzed tumors. Finally, some imbalance in gender and tumor stage characteristics, but not affecting the overall results, should be explained by the legal norms and the inclusion criteria used in the given study.

## 5. Conclusions

In conclusion, this study based on HPV testing assays and a robust platform of IHC methods used to further explore p16 status and the presence of viral oncoproteins E6/E7 confirmed a high prevalence of HPV16 genotype in laryngeal and hypopharyngeal cancers. The absence of the HPV E6/E7 oncoproteins in some tumor samples suggests a mechanism different from the viral integration tumorigenesis mechanism. Unlike in OPSCC, the application of p16 IHC as a surrogate marker of active HR-HPV infection in LSCC and HPSCC appears impractical.

## Figures and Tables

**Table 1:** Patients’ characteristics.

	Cases (*n* = 72)	
	LSCC (*n* = 41)	HPSCC (*n* = 31)
Sex:		
Male	39	29
Female	2	2
Hazardous habits		
Smoking	37	27
Excessive drinking	8	7
Age (median)	64.3	65.9
T grade:		
T1	4	0
T2	8	4
T3	24	16
T4	5	11
N grade		
N0	35	6
N1	4	16
N2	2	8
N3	0	1
M grade		
M0	40	27
M1	1	4
Stage:		
I	4	0
II	7	0
III	22	10
IV	8	21

**Table 2:** Oligonucleotide primers used for HPV DNA detection.

Primers	Sequence (5′-3′)	Amplicon (bp)
β-globin primers		
GS 268	ACACAACTGTGTTCACTAGC	200
GS 269	TGGTCTCCTTAAACCTGTCTTG	
Consensus primers		
MY09	CGTCC(AC)A(AG)(AG)GGA(T)ACTGATC	450
MY11	GC(AC)CAGGG(AT)CATAA(CT)AATGG	
GP5+	TTTGTTACTGTGGTAGATACTAC	150
GP6+	GAAAAATAAACTGTAAATCATATTC	
Type-specific primers		
16.L1-1	TGCTAGTGCTTATGCAGCAA	152
16.L1-2	ATTTACTGCAACATTGGTAC	
18.1	AAGGATGCTGCACCGGCTGA	216
18.2	CACGCACACGCTTGGCAGGT	

**Figure A1:**
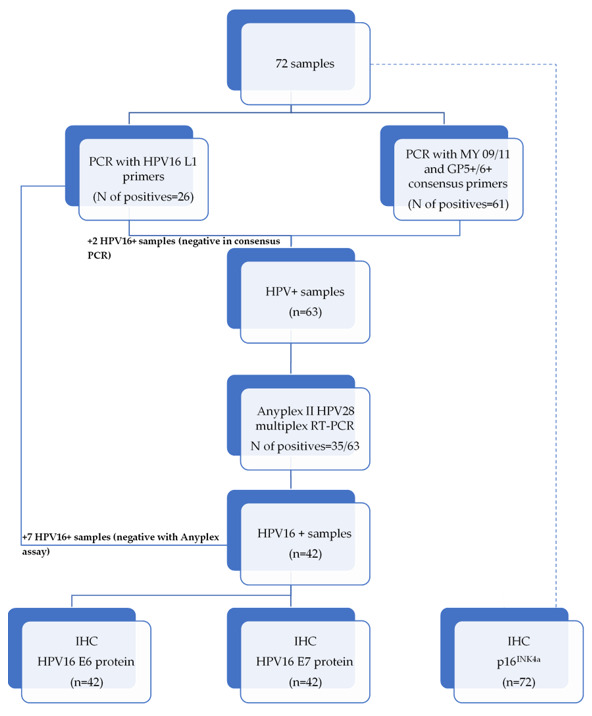
The workflow scheme of the study.

## Supplementary Materials

The following are available online at https://www.mdpi.com/article/10.3390/v13061008/s1, Table S1: The results of different PCRs, Figure S1: Correlation between the Anyplex II HPV 28 assay semiquantitative (viral load) data and viral E6/E7 oncoprotein expression determined immunohistochemically in LSCC and HPSCC tissue samples, Figure S2: Correlation between the p16 IHC results, viral E6/E7 oncoprotein expression determined immunohistochemically, and PCR results in LSCC and HPSCC tissue samples.
