# Supplementary material for: Identification of High-Risk Human Papillomavirus DNA, p16, and E6/E7 Oncoproteins in Laryngeal and Hypopharyngeal Squamous Cell Carcinomas
Source: Viruses. 2021 May 27;13(6):1008. doi: 10.3390/v13061008 (PMC8229053; doi:10.3390/v13061008)
Supplement: Supplementary file 1 [file viruses-13-01008-s001.zip › viruses-1234457-supplementary.pdf]

Nr.	β-globin	MY 09/11	GP5+/6+	HPV16 L1	Anyplex II HPV 28	Anyplex II HPV 28 semiquantitative assessment	p16	Tumor site
1	+	-	+	-	-	-	-	L
2	+	-	-	-	NA	NA	-	L
3	+	-	-	-	NA	NA	-	L
4	+	-	-	+	16	+	+	L
5	+	-	+	+	16	++	-	H
6	+	+	-	-	16	+++	+	L
7	+	-	+	-	-	-	-	L
8	+	-	+	+	16	+	-	L
9	+	-	+	+	16	+	-	L
10	+	-	-	+	16	+	-	L
11	+	-	+	+	16	+	-	L
12	+	-	-	-	NA	NA	-	L
13	+	-	+	+	16, 56	++	-	H
14	+	-	-	-	NA	NA	-	L
15	+	-	+	+	16	++	-	L
16	+	+	+	-	-	-	-	L
17	+	+	-	-	16	+	+	L
18	+	-	+	-	-	-	-	L
19	+	-	+	-	16	+	-	L
20	+	-	+	-	16	+	-	L
21	+	-	+	-	16	+	-	L
22	+	-	-	-	NA	NA	-	L
23	+	-	+	+	16	+	-	L
24	+	-	+	-	16	+	-	L
25	+	-	+	+	-	-	-	L
26	+	-	+	-	-	-	-	L
27	+	+	-	-	-	-	-	L
28	+	+	+	-	-	-	-	L
29	+	-	-	-	NA	NA	-	L
30	+	-	-	-	NA	NA	+	L
31	+	-	+	+	16	++	-	H
32	+	-	+	-	16, 31	+	-	L
33	+	+	-	-	16, 31	+	+	L
34	+	-	-	-	NA	NA	-	L
35	+	+	+	-	16	+	-	L
36	+	+	-	-	16	+	+	L
37	+	-	-	-	NA	NA	-	L
38	+	+	-	-	-	-	-	L
39	+	+	+	-	16	++	-	L
40	+	-	+	-	-	-	-	L

**41**	+	-	+	+	16	+	-	L
**42**	+	-	+	+	16	++	-	L
**43**	+	-	+	-	-	-	-	L
**44**	+	-	+	-	16	+	-	L
**45**	+	-	+	+	16	+	-	H
**46**	+	-	+	-	-	-	-	H
**47**	+	-	+	-	16	+	-	H
**48**	+	-	+	+	16	++	-	H
**49**	+	-	+	-	16	+	-	H
**50**	+	-	+	-	-	-	-	H
**51**	+	-	+	-	-	-	-	H
**52**	+	-	+	-	16	+	-	H
**53**	+	-	+	-	16	+	-	H
**54**	+	+	+	+	16	++	+	H
**55**	+	-	+	+	-	-	-	H
**56**	+	-	+	-	-	-	-	H
**57**	+	-	+	-	-	-	-	H
**58**	+	-	+	+	-	-	-	H
**59**	+	-	+	-	-	-	-	H
**60**	+	-	+	+	-	-	-	H
**61**	+	-	+	-	-	-	-	H
**62**	+	-	+	-	-	-	-	H
**63**	+	-	+	+	-	-	-	H
**64**	+	-	+	-	-	-	-	H
**65**	+	-	+	+	16	+++	+	H
**66**	+	-	+	+	-	-	-	H
**67**	+	-	+	+	-	-	-	H
**68**	+	-	+	+	16	+	-	H
**69**	+	-	+	+	16	+	-	H
**70**	+	-	+	-	-	-	-	H
**71**	+	-	+	+	16	++	-	H
**72**	+	-	+	-	-	-	-	H

Table S1. The results of different PCRs. Description of grades: “-“ – negative, “+” – positive, “NA” – not available (PCR was not performed), “L” – LSCC sample, “H” – HPSCC sample; in Anyplex II HPV 28 semiquantitative assessment the viral load was graded as follows: “+” – low, “++” – moderate, and “+++” – high.

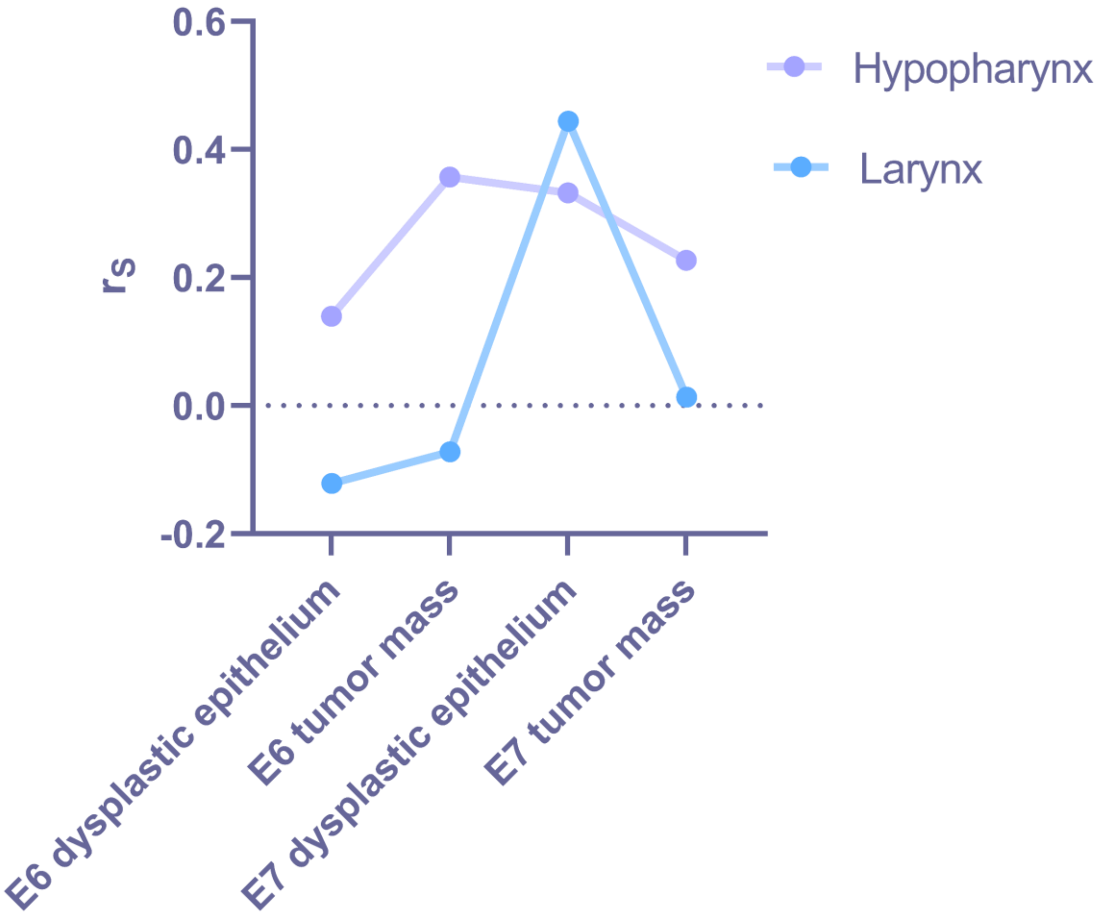

Figure S1. Correlation between the Anyplex II HPV 28 assay semiquantitative (viral load) data and viral E6/E7 oncoprotein expression determined immunohistochemically in LSCC and HPSCC tissue samples. P values for **r**s in HPSCC tissue samples are 0.584, 0.122, 0.189, and 0.335 for E6 oncoprotein in the dysplastic epithelium, E6 oncoprotein in tumor mass, E7 oncoprotein in the dysplastic epithelium, and E7 oncoprotein in tumor mass, respectively. Each dot represents the correlation coefficient; blue dots represent laryngeal tissue samples, and violet dots – hypopharyngeal tissue samples. Correlations determined by Spearman's correlation test.

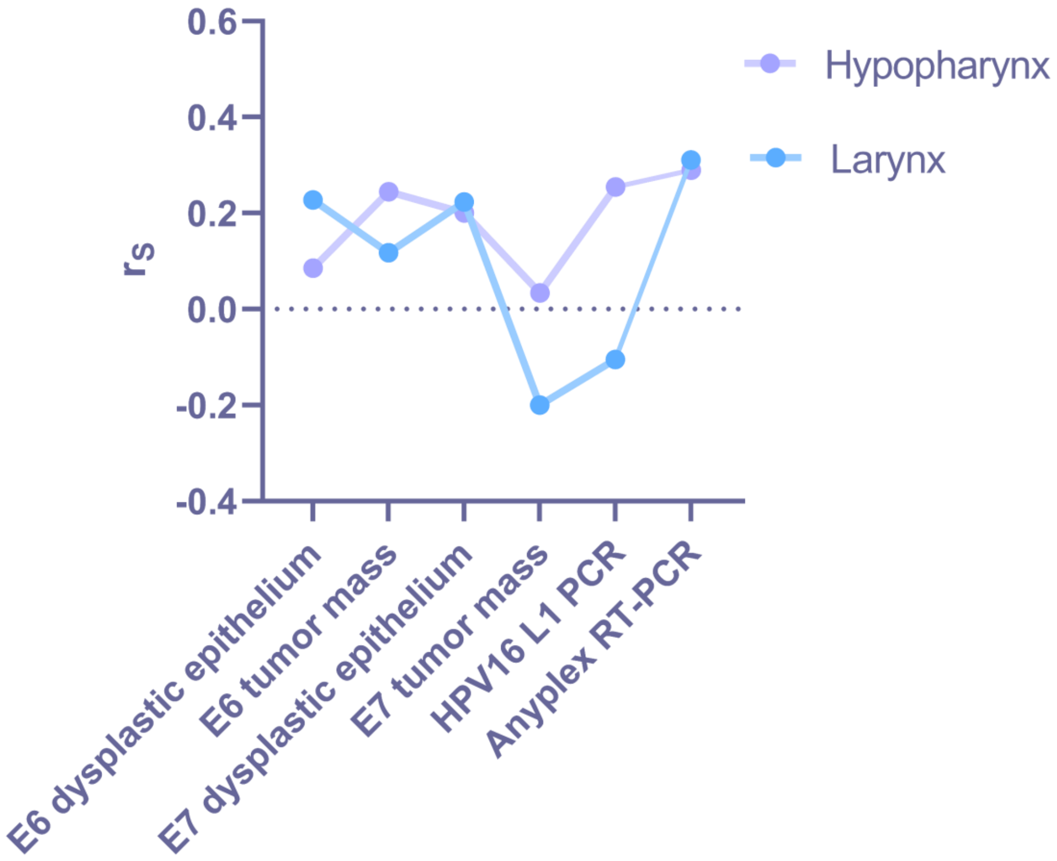

Figure S2. Correlation between the p16 IHC results, viral E6/E7 oncoprotein expression determined immunohistochemically, and PCR results in LSCC and HPSCC tissue samples. P values for **r**s in **LSCC** tissue samples are 0.320, 0.600, 0.357, 0.386, 0.569, and 0.083 for E6 oncoprotein in the dysplastic epithelium, E6 oncoprotein in tumor mass, E7 oncoprotein in the dysplastic epithelium, E7 oncoprotein in tumor mass, HPV16 L1 PCR, and Anyplex RT-PCR, respectively. P values for **r**s in **HPSCC** tissue samples are 0.735, 0.299, >0.999, 0.889, 0.168, and 0.114 for E6 oncoprotein in the dysplastic epithelium, E6 oncoprotein in tumor mass, E7 oncoprotein in the dysplastic epithelium, E7 oncoprotein in tumor mass, HPV16 L1 PCR, and Anyplex RT-PCR, respectively. Each dot represents the correlation coefficient; blue dots represent laryngeal tissue samples, and violet dots – hypopharyngeal tissue samples. Correlations determined by Spearman's correlation test.
